# From conformity to loss of control: psychological mechanisms in Suárez’s biting incidents and the development of the SDA model

**DOI:** 10.3389/fpsyg.2025.1670490

**Published:** 2025-12-04

**Authors:** Zhao Zhang, Shaoran Yu

**Affiliations:** 1 School of Sports Training, Chengdu Sport University, Chengdu, China; 2 Deyang Vocational College of Technology and Trade, Deyang, China

**Keywords:** football athletes, unsanctioned aggression, self-control model, reversal theory, ego depletion, stress-depletion-aggression model (SDA)

## Abstract

Current studies on Luis Suárez’s three biting incidents in football reveal considerable shortcomings: an emphasis on discrete occurrences, disregard for dynamic psychological processes, and dependence on initial trait attribution. These deficiencies hinder the elucidations of recurrent unauthorised aggression by non-habitual aggressors in high-pressure situations. This research fills this need through case analysis and theoretical synthesis. Using biting occurrences as case studies, it amalgamates the self-control model and reversal theory to develop the stress-depletion-aggression model (SDA). The model outlines a three-stage hostility trajectory: (1) Internal and external stressors systematically diminish self-control resources through physiological and cognitive mechanisms; (2) Continuous depletion undermines inhibitory control and alters motivational priorities; (3) A transition in meta-motivational states (from conformist to negativistic + telic/mastery + telic) incites unsanctioned aggression. The findings indicate that the SDA model accomplishes multilevel integration of physiological, neurocognitive, and motivational factors; recognises ego depletion as the primary mediator between stress and motivational transitions; and improves predictive capability for unsanctioned aggression beyond conventional theories. The study indicates that unsanctioned aggression arises from the interactive effects of stress accumulation, resource depletion, and significant motivational-state shifts, establishing a scientific foundation for anticipating athlete hostility, formulating remedies, and optimising competition regulations. The proposed SDA model represents a novel theoretical integration, systematically combining the physiological-cognitive mechanisms of self-control resource depletion with the dynamic reversals of meta-motivational states, thereby providing a multi-level, testable framework for understanding and predicting sudden, unsanctioned aggression by non-habitual aggressors under high pressure.

## 1 Introduction

In a 2014 FIFA World Cup game, Luis Suárez bit another football player’s shoulder after a positional confrontation. This event swiftly garnered international media attention and stimulated extensive discourse on social media platforms.

## 2 Background of Suárez

Born in Salto, Uruguay, in 1987, Suárez grew up in a single-parent family with seven siblings, receiving sole support from his mother. He began his professional football career as a striker at the age of 14 and subsequently played for prestigious European clubs, including Ajax, Liverpool, Barcelona FC, and Atlético Madrid. Throughout his career, he has secured multiple team championships and individual accolades, establishing himself as a world-class footballer. In 2025, he competes for Inter Miami CF in Major League Soccer (MLS).

## 3 Chronology of Suárez’s biting incidents

### 3.1 Amsterdam, Netherlands (November 2010)

In the fifteenth round of the Eredivisie season, Ajax, with Suárez, played to a 0–0 draw against PSV Eindhoven. As the match neared its end, a brawl ensued between players from both teams after Ajax midfielder Rasmus Lindgren received a red card. During this altercation, Suárez—then Ajax captain—abruptly bit PSV midfielder Otman Bakkal on the left collarbone. Within this chaotic and high-arousal environment—a classic pressure scenario—Suárez’s action can be interpreted as a critical failure of self control. Despite Bakkal presenting the injury to the referee, no immediate sanction was imposed. The Royal Dutch Football Association subsequently imposed a seven-match suspension.

### 3.2 Liverpool, England (April 2013)

During a Premier League match against Chelsea, with Liverpool trailing 1–2 in the 65th minute, Suárez received possession in the opposition penalty area but failed to bypass defender Branislav Ivanović. This moment of personal failure in a high-leverage situation, compounded by the ongoing pressure of chasing a game against a direct rival, likely represented a significant spike in frustration and ego depletion. In the subsequent physical contest, Suárez bit Ivanović’s right upper arm following loss of possession. This rapid transition from competitive engagement to overt aggression suggests a sudden motivational reversal, where the accumulated pressure and depletion overwhelmed his capacity for conformist, rule-compliant behaviour. Although the match official imposed no disciplinary measures, the Premier League subsequently issued a ten-match suspension.

### 3.3 Natal, Brazil (June 2014)

During the pivotal Group D encounter between Uruguay and Italy at the 2014 FIFA World Cup, with the scoreline at 0–0 in the 79th minute, Suárez bit Italian defender Giorgio Chiellini’s left shoulder within the penalty area. This incident, occurring at cumulative stress of a scoreless, peak of global sporting pressure, likely exacerbated by his personal goalless performance, seemingly depleted his self-control resources to a critical threshold. Chiellini immediately exhibited bite marks to the referee, who administered no sanction during play. Two days after the match, FIFA’s Disciplinary Committee enacted a nine-match international ban, a four-month suspension from all football activities, and a CHF 100,000 fine in accordance with Article 22 of the FIFA Disciplinary Code (People’s Daily Online, 2014).

#### 3.3.1 Additional incident (April 2025)

During the second leg of the April 2025 CONCACAF Champions League quarter-finals, Suárez initiated a biting motion towards his teammate Jordi Alba’s finger, making momentary contact, but aborted the action abruptly.

As of 2023, Suárez had amassed 731 official appearances for club and country, netting 453 goals and earning a solitary red card. A comparative examination with current best forwards—Lionel Messi (2 red cards/870 appearances), Cristiano Ronaldo (11/963), and Neymar Jr. (9/493)—illustrates his infrequent commission of significant fouls. His personal awards, such as Dutch Footballer of the Year, Premier League Player of the Season, and the European Golden Shoe, further demonstrate his remarkable professionalism. The repetition of three isomorphic biting episodes under similar high-pressure situations presents a distinctive framework for examining aggressiveness triggers in professional footballers, offering substantial research significance.

### 3.4 Concise overview

An examination of Luis Suárez’s three historically and geographically diverse biting events uncovers five recurring patterns:

(1) Match Criticality: ① Eredivisie: Ajax vs. PSV (a traditional “Big Three” derby alongside Feyenoord), generating intense competitive motivation; ② Premier League: Liverpool vs. Chelsea (first versus second in league standings), widely perceived as a decisive title decider; ③ FIFA World Cup: Uruguay vs. Italy (final group stage match requiring victory for knockout qualification).
(2) Temporal Occurrence: All events occurred during the latter half (90 + minutes, 65th minute, and 79th minute, respectively).
(3) Competitive Disadvantage: Suárez’s team was not in the lead at each incident: ① Ajax 0:0 PSV; ② Liverpool 1:2 Chelsea; ③ Uruguay 0:0 Italy.
(4) Performance Deficiency: As the principal striker, Suárez did not score in any pivotal matches and committed a handball that directly led to a penalty, exacerbating his team’s disadvantage.
(5) Sanction Severity: Each event led to exceptional post-match penalties (7–10 matches), significantly above the typical 3-match ban for red-card infractions—despite the absence of in-match disciplinary measures.

Although injurious conduct occurs regularly in football, biting represents an exceptionally rare phenomenon. Its infrequency resulted in ambiguous regulatory prohibitions during Suárez’s incidents. Biting constitutes unsanctioned aggression by transgressing reasonable competitive boundaries, simultaneously violating competitive integrity and sports ethics (Martínková and Parry, 2015). Whilst previous investigations have examined Suárez’s World Cup incident through legal, sociological, and related frameworks, the present pattern analysis reveals his three biting incidents were neither isolated nor inadvertent. These episodes consistently occurred during critical match phases, particularly in the second half when Suárez was at a competitive disadvantage and scoreless, representing unsanctioned aggression that led to sanctions significantly exceeding conventional disciplinary thresholds.

Therefore, extant research exhibits three notable limitations:

(1) They primarily examined a single incident, failing to systematically synthesise and dissect all three biting events (Davis and Ryall, 2017; Mills and Boardley, 2016).
(2) They focused on post-hoc accountability and media impact, neglecting dynamic psychological progression within match contexts (Borge et al., 2015; Islam and Jones, 2014).
(3) There is a dependence on trait-based attribution, which fails to explain the recurrence of aggressive behaviour among non-habitual aggressors when they are under pressure (Anderson and Bushman, 2002; Kerr, 2004).

This study contends that addressing these limitations necessitates the convergence of two theoretical frameworks: the self-control model and reversal theory. The former clarifies that continuous pressure diminishes cognitive resources, hindering behavioural control (Muraven et al., 1998), whilst the latter describes how shifts in meta-motivational states lead to unauthorised aggression (Apter and Heskin, 2001). We present the stress-depletion-aggression (SDA) model, a novel theoretical integration that systematically deconstructs multi-level aggression mechanisms using a “motivation-resource” dual-pathway framework. This methodology seeks to enhance preventative and control measures for athlete aggression.

This study employs an in-depth single-case analysis, focusing on the repeated biting incidents of Luis Suárez. This research design holds unique advantages in the initial stages of theory building. A “critical case”—that is, a case where the phenomenon is manifested in its most pronounced and purest form under extreme conditions—can act as a “social microscope” clearly revealing complex psychological mechanisms difficult to observe in ordinary situations (Nickels et al., 2022). The unique value of the Suárez case lies in its “perfect storm” characteristics: a recognised non-habitual aggressor with only one red card in over 700 professional matches, who nevertheless exhibited three highly consistent episodes of extreme aggression under specific high-pressure-depletion-reversal conditions. This extreme contrast and clear pattern provide unparalleled clarity and explanatory power for constructing a theoretical model about state loss of control rather than trait aggression.

## 4 Biting and aggressive behaviour in sports competitions

Although match officials imposed no in-game sanctions for Luis Suárez’s three biting incidents, all resulted in substantial post-match suspensions. Michel D’Hooghe, Chairman of the FIFA Medical Committee and Executive Committee member, explicitly characterised biting behaviour as violating football’s conventional norms and constituting an exceptionally severe disciplinary case.

In 2018, FIFA amended World Cup regulations to explicitly prohibit biting for the first time. Under revised Article 12, biting now warrants equivalent sanctions to other serious misconduct: immediate dismissal via red card and a direct free kick. Media discourse subsequently termed this regulation—catalysed by Suárez’s incident—the “Suárez Clause.”

### 4.1 Conventional categorisation of aggressive behaviour

Aggressive behaviour refers to any act intended to harm or injure another living being (physically, psychologically, or verbally) who is motivated to avoid it (Baron and Richardson, 1994; Benjamin, 2015).

In competitive sports, this behaviour generally falls into two categories (Bar-Eli et al., 2015):

(1) Hostile Aggression: Emerges from rage or a perceived lack of control, with the explicit intention to inflict harm. In a 2006 Premier League match, Jermain Defoe bit Javier Mascherano’s arm in the 42nd minute as an immediate retaliation to numerous fouls. Contrasts with Suárez’s episodes manifest in three dimensions: context of provocation (repeated fouls versus unprovoked actions), emotional intensity (high versus comparatively lesser), and timing of occurrence (first half versus late second half).
(2) Instrumental Aggression: Driven by strategic aims (e.g., tactical disruption), when violence serves as a means to competing ends, generally avoiding significant injury. Tactically, fouling interrupts opposing attacks. However, Suárez bit opponents twice during open play whilst his team possessed the ball and once during a stoppage. These incidents lacked clear competitive objectives, suggesting motivations beyond straightforward categorisation.

Scholars observe that whilst both hostile and instrumental aggression involve intent to harm, they differ fundamentally: hostile aggression typically manifests reactively from anger following provocation, whilst whilst instrumental aggression usually does not involve anger and is focused on achieving specific goals (Bushman and Huesmann, 2010). Crucially, an action’s permissibility varies by sport—tackling permitted in rugby constitutes foul play in football. Kerr (2004) consequently defined sporting aggression through rule compliance: *Sanctioned aggression* referred to acts within written rules or accepted norms, potentially providing gratification; *Unsanctioned aggression* referred to acts exceeding rule boundaries or violating sport ethics. Whilst this framework addresses limitations of the hostile/instrumental dichotomy, two controversies persist: its inability to explain hybrid aggression combining both motives simultaneously and arguments that all aggression serves instrumental goals (Grange and Kerr, 2010).

Consequently, competitive sports research increasingly prioritises tracing motivations underlying rule-violating behaviours (Conway et al., 2024), necessitating a rigorous analysis of hybrid meta-motivational complexes.

### 4.2 Overview of reversal theory

Reversal theory (Apter and Heskin, 2001) was initially developed to analysis individual behavioural motivations, with particular emphasis on the driving mechanisms underlying specific activities. Its core tenets posit that (1) Motivation exhibits a binary oppositional structure comprising four pairs of meta-motivational states; (2) These states dynamically reverse—with only one state per pair active at any time—contingent upon situational demands. The four state pairs include Telic-Paratelic, Conformist-Negativistic, Mastery-Sympathy, and Autic-Alloic (Table 1).

**Table 1:** Core behavioural characteristics of the 8 meta-motivational states.

Meta-motivational State	Behavioural characteristics	Typical example
Telic	Serious, highly planned, outcome-orientated; pursues efficient achievement of training goals; often accompanied by low arousal.	A goalkeeper evaluates an adversary’s penalty kick tendencies to devise a save strategy.
Paratelic	Spontaneous, impulsive, sensation-seeking; the core focus is experiencing the fun of sport; prefers high arousal.	A skateboarder attempting an unpractised high-altitude flip manoeuvre.
Conformist	Agreeable, cooperative; strictly adheres to written rules and industry norms.	A football player intentionally kicks the ball out of bounds to permit an injured opponent to obtain medical assistance.
Negativistic	Rebellious, stubborn; reactive opposition to perceived oppressive situations.	A basketball player performs a “shushing” gesture towards provocative spectators.
Mastery	Highly competitive; seeks dominance and control; aims to suppress opponents through assertive performance.	A boxer utilises an assertive offensive approach to undermine the opponent’s resistance.
Sympathy	Values harmony and empathy; proactively builds emotional connections.	A badminton player assists an opponent stretching when he/she suffers a cramp.
Autic	Focuses on personal feelings and interests; actions primarily satisfy the self.	A football player persists in attempting individual dribbles despite their team’s significant deficit.
Alloic	Concerned with others’ emotions and achievements; exhibits altruism and collective pride; derives pleasure from others’ success.	A volleyball player experiences greater exhilaration from assisting a teammate in executing a spike than from scoring one independently.

The operation of the meta-motivational state is governed by three principles: (1) Mutual exclusivity: At any given time, only one state within a pair may be active; (2) Dynamic reversibility: Transitions between states are prompted by triggers such as contingency, frustration, or satiation; (3) Context dependency: The activation of a state is fundamentally reliant on the individual’s assessment of their environment.

#### 4.2.1 Reversal theory classification of aggressive behaviour

Reversal theory offers a robust explanatory framework for sports aggression, characterised by three principal advantages: (1) Accommodation of anger-independent aggression; (2) Prioritisation of proximal contextual factors (e.g., referee decisions, scoreboard pressure) over distal influences (e.g., genetic predispositions, cultural norms); and (3) Integration of mixed meta-motivational drivers, thereby circumventing single-cause attribution limitations.

**Figure 1:**
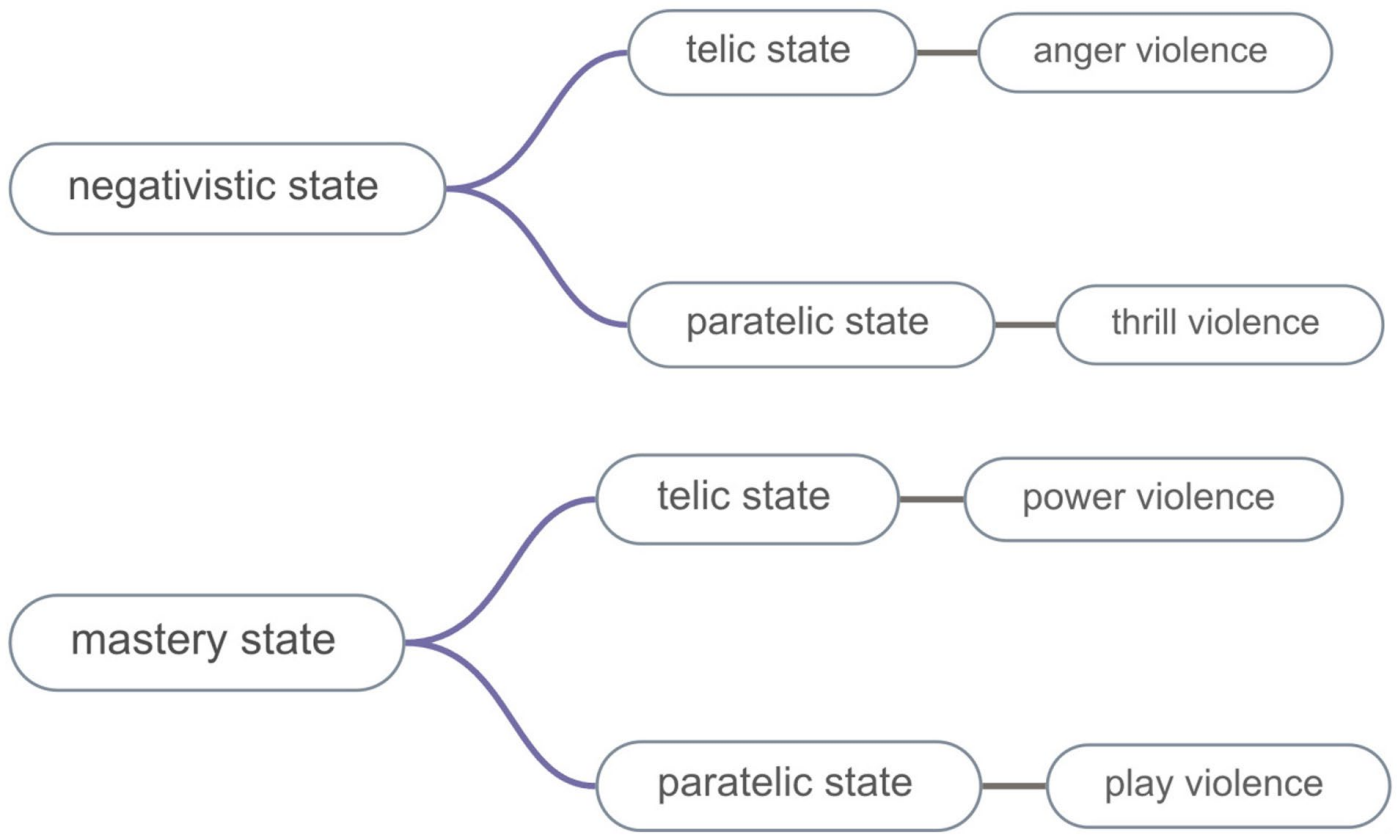
Showing the meta-motivational state combinations which underlie the different types of violence.

The theory’s primary strength lies in elucidating how meta-motivational states’ reversals dynamically modulate aggressive behaviour. Apter delineates four states pertinent to athletic aggression: Negativistic (rule-defiant), Mastery (dominance-orientated), Telic (goal-directed), and Paratelic (sensation-driven). Dynamic transitions between these states may precipitate abrupt behavioural shifts—from strategic execution to emotional outbursts (Kerr, 2021; Roberts et al., 2020; Table 2). This approach has proven effective in qualitative investigations examining athletes’ paradoxical aggressive motivations (Hudson et al., 2016; Grange and Kerr, 2010; Ullah and Iftikhar, 2021) (see Figure 1).

**Table 2:** Four types of aggressive behaviour based on meta-motivational state combinations.

Aggression type	State combination	Core motivation	Emotional arousal	Typical behaviour
Anger	Negativistic + Telic	Retaliation/Venting	High anger	Striking an opponent after being provoked
Thrill	Negativistic + Paratelic	Sensation-Seeking	High excitement	Unnecessary provocation whilst leading
Power	Mastery + Telic	Deterrence/Control	Calm	Intentionally injuring/threatening an opponent
Play	Mastery + Paratelic	Enjoying	Excitement	Legal physical challenges, tackles, collisions

Reversal theory classifies aggression according to regulatory compliance: (1) Sanctioned aggression (play type): Adherence to rules and ethics, constituting ethically permissible competitive conduct; (2) Unsanctioned aggression (anger, thrill and power types): Violation of regulatory and ethical boundaries, typically incurring post-event sanctions.

Conventional aggression frameworks (e.g., hostile/instrumental dichotomy; frustration-aggression theory) provide fundamental classifications (Kerr, 2004) but rely on static attributions inadequate for elucidating dynamic meat-motivational progressions in Suárez’s case. Key explanatory limitations include: Hostile aggression’s failure to align deterrent aims with minimal anger; Instrumental aggression’s oversight of non-tactical symbolic assaults; Frustration-aggression theory’s inadequacy regarding non-frustration-driven thrill-seeking.

Reversal theory addresses these constraints through dynamic combinations and reversals of meta-motivational states.

The theory posits two aggression mechanisms:

(1) Synergistic mechanism: Couples mastery (power demonstration) and telic (deterrence objective) states in power aggression;
(2) Reversal mechanism: Triggers critical transitions from conformist to negativistic states under pressure.

This paradigm resolves key limitations of classical models whilst augmenting the self-control resource model through interconnected pathways:

Meta-motivational pathway (reversal theory): Dynamically generates aggressive impulses.
Resource pathway (ego depletion): Mediates behavioural inhibition failure.

Together, they establish a dual-pathway “motivation-resource” framework enabling targeted interventions for aggression.

It is necessary to preface that the theoretical analysis of Suárez’s psychological states in this section constitutes a retrospective psychological construction based on situational logic. As real-time interviews or psychological measurements of the individual involved in the events were not possible, our analysis is strictly grounded in three chains of publicly verifiable information: (1) objective match situational data; (2) Suárez’s observable on-field behaviours; (3) his long-term career behavioural baseline. Using principles from self-control theory and reversal theory, we integrate these scattered behavioural “clues” into a coherent psychological narrative. The aim is to demonstrate the explanatory potential of the SDA model, not to make an irrefutable definitive statement about Suárez’s internal world. This analytical paradigm is widely used in research on psychological mechanisms in historical cases, its value lying in generating testable hypotheses rather than providing ultimate proof.

## 5 Theoretical analysis of Suárez’s biting episodes: an integrated SDA model incorporating self-control and reversal theory

A comprehensive synthesis of multidisciplinary perspectives—including legal, communicative, medical, and macro-psychodynamic approaches—reveals the complex aetiology of Suárez’s biting incidents. However, these frameworks have significant limitations because they primarily emphasise *post hoc* accountability or distal causation whilst neglecting the real-time psychological mechanisms that occur during the progression of a match. The central theoretical challenge remains unresolved: why would an elite non-habitual aggressor repeatedly resort to extreme biting behaviour under specific high-pressure conditions? Addressing the question requires precise examination of psychological states at the behavioural threshold, focusing on two interconnected mechanisms: (1) Depletion of self-control resources under sustained pressure, culminating in inhibitory failure; (2) Critical meta-motivational state reversals induced by ego depletion, subsequently activating aggression.

Integration of these mechanisms establishes a dynamic model explaining recurrent unsanctioned aggression.

### 5.1 Ego depletion: the failure mechanism of aggression inhibition

#### 5.1.1 Physiological and cognitive foundations of the self-control model

Self-control theory examines aggression through the lens of ego depletion—the exhaustion of self-regulatory resources. Self-control denotes an individual’s capacity to suppress innate impulses, defer immediate gratification for long-term objectives, and adhere to social norms (Baumeister et al., 2018). This regulatory faculty functions as a limited resource that restrains self-serving behaviour. The self-control resource model asserts that self-regulation relies on limited psychological resources. The allocation of these resources to previous tasks reduces self-control ability, a condition referred to as ego depletion (Englert, 2017). As a result, ego depletion diminishes performance on later self-regulatory activities.

Chronic stress and cognitively intensive regulation could enhance prefrontal glucose metabolism, exhausting self-control reserves. This depletion markedly correlates to increased hostility in teenagers (Nie et al., 2018). Laboratory research indicates that persons experiencing ego depletion show significantly increased likelihood of aggressiveness after provocation (Baumeister et al., 2024). In contrast, glucose replenishment significantly reduces subsequent aggression in individuals experiencing depletion (Denson et al., 2010; Thériault and Dandeneau, 2023). Intervention studies yield compelling evidence: experimental groups undergoing 2 weeks of self-control training exhibited markedly fewer aggressive responses than control groups (Denson et al., 2011).

In the field of cognitive neuroscience, Inzlicht et al. (2016) proposed two models of ego depletion: the Shifting Priorities Model and the Valuation Model. The previous framework redefines self-control as a dynamic motivational trade-off process in which prolonged self-regulation heightens task aversion, thereby altering motivational priorities from long-term goal commitment (“have-to” goals) to the pursuit of immediate satisfaction (“want-to” goals). In contrast, the Valuation Model conceptualises self-control as a value-driven decision-making process, wherein the ventromedial prefrontal cortex synthesises incoming signals to create subjective value representations that influence behaviour (Rangel and Hare, 2010; Ting et al., 2023). Both models agree on a key idea: aggression might happen when our motivation changes and we misjudge the value of our actions—specifically, when we think the immediate benefits of violence are greater than they really are and overlook its negative consequences. Thus, the motivation system emphasises high-arousal, low-effort violent behaviours.

This research clarifies the regulatory mechanism of self-control: a preserved self-control capacity suppresses aggressive urges, thereby diminishing aggression. Ego depletion diminishes inhibitory control over impulses, leading to increased violence.

The General Aggression Model (GAM) and I^3^ Model comprehensively clarify the mechanisms by which self-control influences aggression. As stated by GAM (Anderson and Bushman, 2002), psychological resources predominantly modulate aggression via affecting decision-making processes and outcome assessment. Aggression is more likely to occur when resource-depleted individuals encounter displeasure with outcomes. Research on ego depletion substantiates this mechanism (Barlett et al., 2016): diminished resources hinder the ability to evaluate outcomes, hence markedly elevating the probability of aggressiveness following adverse incidents.

Evidence from sport psychology supports this mechanism. Sofia and Cruz’s (2015) questionnaire study showed inverse correlations between athletes’ self-control capacity and aggression, perhaps mediated by enhanced attentional control that facilitates the blocking of irrelevant thoughts. Englert and Bertrams (2012) further elucidated anxiety’s moderating function: in conditions of weakened self-control, high-anxiety athletes demonstrate decreased task concentration and compromised aggressiveness regulation.

The I^3^ Model (Zhang, 2021) conceptualises self-control as an inhibitory mechanism that diminishes the probability and intensity of behaviour. Its three-part framework consists of:

*Inhibition*: Self-regulatory ability
*Impelance*: Internal behavioural inclination
*Instigation*: Contextual stimuli

Aggression happens when the combined driving forces (Impellance + Instigation) exceed the inhibitory force (Inhibition). Empirical data (Finkel and Hall, 2018) strongly substantiates that Inhibition: (1) predicts less aggression; (2) mitigates the effects of Impellance/Instigation. Consequently, self-control mitigates violent inclinations and counteracts situational provocation, thereby effectively restricting violence.

Suárez’s biting behaviour is consistent with a pattern that may be explained by inhibition failure due to ego depletion caused by internal and external demands. All occurrences transpired during high-pressure matches that imposed continuous cognitive demands. As principal striker, Suárez may experienced substantial external pressure from unmet scoring expectations (0 goals in 3 matches; 1 penalty conceded) and internal strain from performance deficiencies. These stressors likely provoked significant anxiety. Prolonged physical exertion impaired self-regulation capacities—including focus maintenance, rule compliance, and emotional control. Resource depletion shifted meta-motivational priorities toward immediate gratification, causing inhibitory failure at critical junctures. Biting—a low-effort, high-arousal act—may reflect potential physiological factors (e.g., dentition morphology) and reinforcement history.

Ego depletion constitutes the core mediator across models, characterised by insufficient resources and inhibitory failures. This mechanism mediates unsanctioned aggression when coupled with pressure-induced anxiety.

#### 5.1.2 Counterfactual analysis and ruling out alternative explanations

The fact that Suárez committed only three biting incidents throughout his career of over 700 high-level matches itself constitutes strong counterfactual evidence: if he were a habitual aggressor driven by stable personality traits, aggressive acts would be more frequent and dispersed, rather than highly concentrated in specific “perfect storm” scenarios of high-pressure-depletion-reversal. His career record of only one red card further refutes an “aggressive personality” as the primary explanation.

Secondly, cultural background also lacks sufficient explanatory power here. Whilst Suárez is from Uruguay, a football culture known for its toughness, biting is an extreme act rare in South American and global football, not a common behavioural pattern among players from that culture. Therefore, cultural factors cannot adequately explain the specificity and repetitive pattern of his behaviour.

In summary, only the SDA model can systematically explain why an elite athlete who maintained high professional discipline most of the time resorted to such specific and consistent unsanctioned aggression only in the few extreme situations where internal and external pressures accumulated to a critical point, self-control resources were nearing exhaustion, and accompanied by a specific reversal of meta-motivational states. The model rules out singular trait or cultural determinism, confirming the centrality of a dynamic, state-process oriented interactive mechanism.

### 5.2 Reversal theory: the dynamic evolution of aggressive motivation

Within reversal theory’s classification paradigm, Suárez’s three biting incidents constitute definitive unsanctioned aggression. These acts violated both codified regulations and athlete-upheld behavioural standards. Three contextual factors demonstrate exceptional severity:

(1) Absence of personal animosity toward victims;
(2) Regulatory omission of specific biting prohibitions contemporaneously;
(3) Unprecedented post-match sanctions imposed by governing bodies.

Collectively, these differentiate biting from conventional fouls (e.g., intentional kicking) through heightened transgression severity.

Biting demonstrates calculated intent and significant harm potential. Unlike typical sports injuries, it presents unique hazards: breached skin facilitates pathogen transmission whilst compromising biter oral health, fundamentally violating athletic risk parameters.

This behaviour constitutes a foundational violation of athletic conventions, exceeding acceptable competitive boundaries and breaching sports ethics. Consequently, it is rejected by the sporting community; its occurrence in globally prominent football compromises institutional integrity and risks public stigmatisation.

Biting violates socio-cultural taboos through associations with predatory behaviour. Legally classified as assault in numerous jurisdictions, it evokes subtexts of dehumanisation. This amalgamation of physical violence and psychological revulsion undermines civilised conduct and standards, causing dual physical/symbolic traumas to societal norms.

These dimensions—ethical transgression, health/safety compromise, and civilisational boundary violation—establish biting as an exceptionally severe unsanctioned assault in professional sports.

#### 5.2.1 Dynamic integration mechanism of meta-motivational states

Reversal theory posits that unsanctioned aggression emerges from dynamic combinations of meta-motivational states that change depending on the situation. Suárez’s biting behaviour likely involved the following state synergies:

##### 5.2.1.1 Mastery + telic combination (power aggression)

It can be characterised as a form of aggressive intimidation that asserts dominance. During critical match phases, Suárez—facing defensive pressure as principal striker—employed illicit aggression to signal deterrence and reassert temporal control. The contest’s goal-orientated nature imbues this behaviour with deliberate intention, distinguishing it from purely emotional reactions. This synergy generated dominance through non-injurious physical pressure for psychological suppression, intrinsically linked to competitive objectives.

##### 5.2.1.2 Negativistic + telic combination (anger aggression)

Goal-driven athletes exhibit negativistic reactions to provocation, eliciting impulsive fury. Suárez’s biting during high-intensity phases constituted adverse responses to perceived rule violations. The 2013 and 2014 incidents likely originated from physical provocations during play, triggering retaliatory reactions amplified by outcome pressure.

Consequently, Suárez’s biting can be interpreted as reflecting a dual motivation: Power aggression prevailed as the strategic aim, but anger aggression served as an immediate catalyst. This behaviour corresponds with the fundamental requirements of unsanctioned aggression: rule transgressions and non-competitive motivation (e.g., deterrence, provocation; Kerr, 2021). Its malevolent nature fundamentally subverts regulatory systems and norms of sportsmanship, transcending mere physical injury intentions.

The meta-motivational combinations offer a comprehensive framework for unsanctioned aggression, with the pivotal shift from potential motivation to behavioural enactment in Suárez’s case stemming from ego depletion that induced state reversals. The ongoing depletion of resources disturbed meta-motivational balance, leading to swift transitions to aggressive meta-motivational states.

#### 5.2.2 Effects of ego depletion and the critical reversal of meta-motivational states

This progression occurs through five sequential phases:

(1) Initial match phase (sanctioned state): Suárez initially operated within sanctioned behavioural parameters (e.g., regulated physical contests, foul avoidance), potentially influenced by conformist + telic or sympathetic + telic state combinations facilitating rule-compliant tactical execution.
(2) Pressure accumulation phase: Escalating external pressure (approaching critical thresholds) and internal pressure (performance deficits) heightened cognitive load and impaired self-regulation capacities. This precipitated meta-motivational reprioritisation, substantially lowering the threshold for conformist-to-negativistic state transitions.
(3) Value reconfiguration phase: Progressive depletion of self-regulatory resources mediated meta-motivational restructuring via dual mechanisms—inhibitory pathway failure and subjective value distortion. This manifested as reduced impulse control, heightened valuation of immediate goals, and diminished prioritisation of long-term compliance objectives.
(4) Reversal threshold reduction: Previously established reversal thresholds requiring significant provocation were compromised by ego depletion, enabling negativistic state activation through minor disputes.
(5) Formation of aggression pathways: When state combinations were altered to negativistic + telic or mastery + telic, biting—distinguished by minimal cognitive expense and significant perceived immediate benefit (e.g., intimidation and pressure release)—was chosen as an effective technique for resolving deadlocks.

### 5.3 Integration of self-control theory and reversal theory: the stress-depletion-aggression (SDA) model

**Figure 2:**
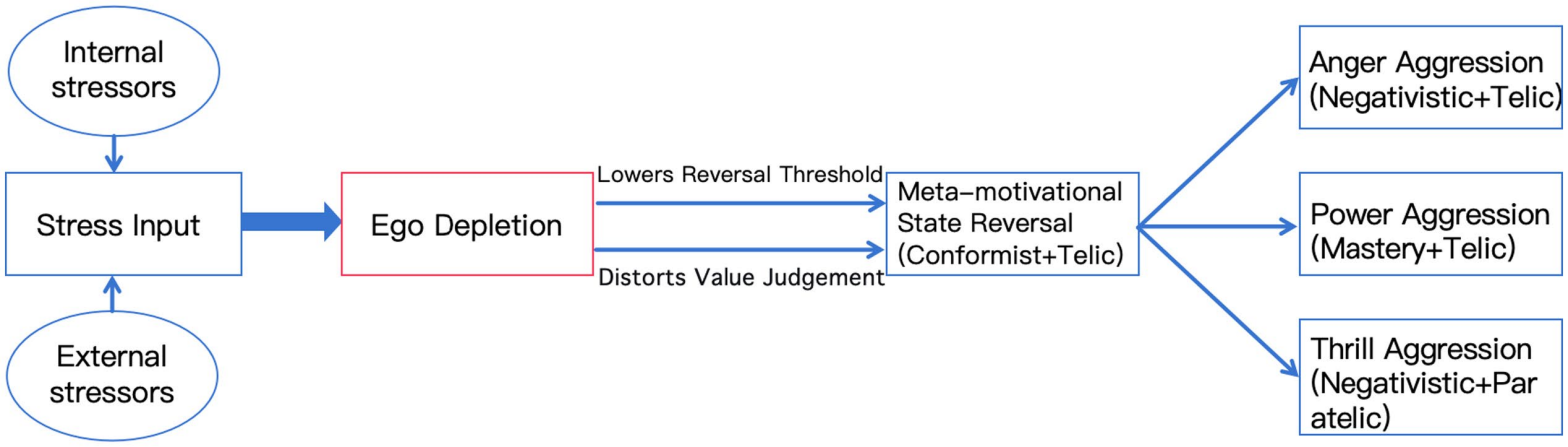
The stress-depletion-aggression (SDA) model. Individual differences and situational factors may moderate the above pathways.

By conducting a thorough investigation of Suárez’s biting events, we combine fundamental principles of self-control theory and reversal theory to create the SDA Model—a triadic dynamic framework elucidating athlete aggressiveness, whose core pathway is illustrated in Figure 2.

This model asserts that violent behaviour arises from the interplay of increasing pressure, diminishing self-control resources, and significant shifts in meta-motivational states, which facilitates prediction accuracy. The framework progresses methodically through three stages: Stress input, ego depletion, and meta-motivational reversal leading to aggressive output.

#### 5.3.1 Stress input: the starting point of dynamic accumulation

Pressure embodies the primary psychological difficulty in athletic competition and serves as the foundation of the SDA model. The fundamental mechanism entails the ongoing depletion of self-control resources by internal and external stressors via physiological, cognitive, and emotional channels.

##### 5.3.1.1 External stressors: match criticality and social expectations

High-stakes matches may activates physiological self-control mechanisms via the HPA axis, elevating cortisol levels and inducing progressive high-arousal states. Researchers suggested that elevated social expectations constitute social-evaluative threats, activating the amygdala and insula, exacerbating anxiety, and competitively consuming attentional and executive control resources (O'Brien et al., 2020; Silvestrini et al., 2020; Wagner and Heatherton, 2016).

##### 5.3.1.2 Internal stressors: performance anxiety and physiological fatigue

We hypothesize that frustration in achieving goals diminishes self-efficacy, triggering mPFC-mediated self-critical rumination and dominance of the default mode network, which directly depletes resources designated for inhibiting aggressiveness. Extended high-intensity exercise could result in muscle lactate buildup, hindering cerebral glucose metabolism. The combination of sympathetic overactivation impairs homeostasis and undermines emotional control (Inzlicht et al., 2016).

Internal and external stressors may synergistically deplete self-control resources through these pathways. When cumulative effects exceed individual tolerance thresholds, non-linear acceleration of resource depletion occurs. Consistent with the Shifting Priorities and Valuation Models, sustained pressure induces behavioural goal-value reweighting: approaching critical thresholds, the subjective valuation of short-term objectives becomes markedly elevated. Consequently, motivational prioritisation shifts from long-term compliance-focused objectives to immediate impasse-resolution strategies.

#### 5.3.2 Ego depletion: progressive resource exhaustion and neural imbalance

Ego depletion constitutes a dynamic process whereby sustained self-regulatory demands exhaust cognitive resources, impairing behavioural control—particularly impulse inhibition (Zha et al., 2025). Critically, motivational priority shifts represent neurophysiologcal accelerated processes that exacerbate depletion through multi-level pathways:

(1) Neurophysiological mechanisms:

Chronic pressure may persistently activates the amygdala whilst inhibiting prefrontal cortex function, biasing resource allocation toward immediate objectives (Guadagno et al., 2021). At depletion thresholds, dorsolateral prefrontal cortex inhibition of the amygdala attenuates, compromising impulse control (Huang et al., 2025). Concurrently, heightened amygdala activation and amplified reward sensitivity to aggressive stimuli occur (Wang et al., 2024).

(2) Cognitive-behavioural mechanisms:

Individuals strategically allocate diminished resources toward motivationally prioritised objectives over obligatory compliance goals, accelerating depletion. During depletion, attentional bias toward provocative cues and preference for low-effort/high-reward options emerge—despite substantial long-term consequences (Inzlicht et al., 2025).

In high-pressure competition, athletes encounter frequent complex decisions and continuous regulatory demands. Rapid resource exhaustion lowers activation thresholds for negativistic and mastery states, substantially increasing aggression probability.

Collectively, ego depletion functions as the core mediator in the SDA model, linking stress input to meta-motivational reversal. Progressive exhaustion levels constitute key antecedents predicting aggression occurrence and typology.

#### 5.3.3 The coupling mechanism from ego depletion to meta-motivational state reversal

Ego depletion and meta-motivational state reversal are not independent processes but are tightly coupled through the following mechanisms, collectively facilitating aggressive behaviour:

(1) Cognitive disinhibition and lowered state reversal thresholds: Depletion of self-control resources may weakens the prefrontal cortex’s inhibitory control over the limbic system. This neural imbalance behaviourally manifests as a sharp increase in the cognitive cost required to maintain a conformist state, whilst significantly lowering the threshold for switching to high-arousal, low-effort states like negativistic or mastery. At this point, a minor provocation or frustration that would be ignored under normal resource levels can become the trigger for state reversal.
(2) Neurocomputational reconfiguration of meta-motivational priorities: According to the valuation model, ego depletion alters the value computation process in the ventromedial prefrontal cortex. The subjective value assigned to immediate rewards is amplified, whilst the consideration of long-term costs is diminished. This reprioritization aligns strongly with a meta-motivational drift from the telic state towards the paratelic state, or a reversal from the conformist state to the negativistic state.
(3) Failure of emotional regulation and stabilisation of high-arousal states: Ego depletion impairs the ability to regulate negative emotions like anxiety and anger. Sustained high arousal creates a psychological environment unfavorable for the calm, serious telic-conformist state, but is highly compatible with the release-seeking negativistic-paratelic state or the control-reclaiming mastery-telic state. Thus, depletion not only permits state reversal but actively promotes the activation and maintenance of those meta-motivational states prone to triggering aggression.

#### 5.3.4 Meta-motivational reversal and aggression output

Reversal theory’s dynamic state reversal mechanism proves pivotal at this stage. Ego depletion elevates aggression probability by lowering meta-motivational state activation thresholds, manifesting as weakened prefrontal inhibition.

With adequate self-regulatory resources, athletes predominantly maintain a conformist state for rule compliance. Upon reaching critical depletion, reduced negativistic state thresholds facilitate the adoption of high-arousal, low-cost mastery strategies for rapid goal attainment, increasing the likelihood of unsanctioned aggression.

The SDA model conceptualises aggression as the product of self-regulatory constraints and dynamic meta-motivational interactions—not isolated events. Its core pathway follows:

Stress input → ego depletion → inhibitory weakening + meta-motivational reprioritisation → significantly elevated aggression probability.
Specific meta-motivational combinations predict aggression typology, requiring dual conditions: ego depletion and specific state activation.

Table 3 details how differential pressure profiles and meta-motivational states explain the four aggression types within the SDA framework.

**Table 3:** Aggression types and their pressure, depletion, and meta-motivational characteristics.

Aggression type	Stressors	Ego depletion level	Meta-motivational combination	Typical case
Anger	External provocation	Medium-High	Negativistic+Telic	Zidane headbutting Materazzi
Thrill	Low-risk situation	Low	Negativistic + Paratelic	Technical foul after scoring a goal
Power	High internal goal pressure	High	Mastery + Telic	Suárez biting
Play	Sanctioned confrontation demand	Low	Mastery + Paratelic	Legal tackles in rugby

The SDA model posits that aggression typology distinctions arise from unique configurations of three core determinants:

(1) Stressor nature: Anger aggression stems predominantly from external provocations, whereas power intimidation-type originates from internal goal-related pressures;
(2) Ego depletion magnitude: Thrill/play manifestations may occur without full depletion, whereas Power/anger types necessitate substantial depletion causing inhibitory failure;
(3) Meta-motivational dynamics: Anger aggression features negativistic intensity surges with telic orientation, contrasting with Play-type’s mastery dominance under paratelic orientation.

The SDA model systematically elucidates Suárez’s biting behaviour: High-pressure situations induce dynamic pressure accumulation, exhausting self-control resources at critical thresholds. This prompts a meta-motivational shift from conformist to negativistic + telic or mastery + telic states, wherein biting—a low-cost, high-reward approach (instant venting/deterrence)—is anticipated as the resultant behavioural output.

Fundamental theoretical contributions of the SDA Model:

Cross-level integration: The initial framework systematically integrates physiological depletion mechanisms (HPA axis, glucose metabolism) and neural inhibitory imbalance (PFC-Amygdala) with the dynamic state reversals of reversal theory, thereby establishing a multilevel physiological-neural-cognitive-motivational framework for aggression.
Dynamic visualisation: Illustrates the nonlinear causal sequence. Pressure leads to depletion, which results in meta-motivational reversal and then triggers aggression output.
Explicit mediation: Identifies ego depletion as the primary mediator connecting pressure to meta-motivational reversal, with the extent of depletion serving as the principal predictor of aggression manifestation.
Predictive enhancement: Defines antecedent-consequent relationships between stressor profiles/depletion levels and state combinations (Table 3), markedly enhancing the prediction of unsanctioned aggression beyond descriptive or attributional theories.

Compared to well-known theories like the General Aggression Model, Frustration-Aggression Hypothesis, and I^3^ Model, the SDA model exhibits enhanced integration depth, dynamic process articulation, mediation elucidation, and predictive efficacy. It offers a thorough theoretical framework for comprehending athletic aggressiveness and facilitates dual-pathway interventions: cognitive and contextual.

## 6 Discussion

This study, through an in-depth analysis of Luis Suárez’s biting incidents, constructs an integrated physiological-neural-motivational stress-depletion-aggression (SDA) model. This model delineates the psychological pathway from pressure accumulation and resource depletion to critical motivational reversal underlying unsanctioned athlete aggression, thereby overcoming the limitations of single-cause attribution characteristic of traditional theoretical frameworks.

The theoretical advancement of the SDA model lies in its targeted resolution of significant limitations within prominent theoretical frameworks (see Table 4), providing a more systematic and empirically testable framework for understanding complex sports aggression.

**Table 4:** Differences between the SDA model and other aggression theories.

Aggression theory	Core perspective	Limitations	Breakthroughs of the SDA model
Hostile/instrumental aggression	Classifies aggression based on motive	Static classification: cannot explain the dynamic mixing and transformation of motives during competition. Ignores resource constraints: does not account for the fact that even with strategic motive, aggression may be inhibited if self-control resources are sufficient	Introduces dynamic mechanisms: explains the dynamic evolution of motives through ego depletion and motivational state reversal. Establishes resource prerequisite: Identifies self-control resources as a key constraint determining whether motive translates into behaviour.
GAM	Emphasises that person-situation interactions influence internal states to elicit aggression	Ambiguous mediating pathways: whilst mentioning internal states, it fails to clearly specify an operational, measurable core mediating variable, particularly the necessary psychological pathway from sustained stress to aggressive behaviour	Identifies core mediator: posits “ego depletion” as the core mediating variable linking sustained stress to aggression, constructing a testable causal chain: Stress → Ego Depletion → Motivational Reversal → Aggression.
I^3^ Model	Aggression results from the interaction of instigation, impellance, and inhibition	Lacks physiological anchors: whilst proposing an interactionist framework, it does not integrate specific physiological mechanisms to quantify the intensity of “Instigation” or its depleting effect on “Inhibition”	Fills physiological gaps: integrates specific pathways including HPA axis activation, cortisol elevation, and inhibition of prefrontal glucose metabolism, enabling dynamic monitoring and quantification of “stress input” and “inhibitory failure.”
Reversal theory	Emphasises the dynamism of meta-motivational states as the basis of aggressive behaviour	Overlooks triggering conditions: whilst describing the phenomenon of state reversal, it inadequately specifies what critical condition triggers the reversal from compliant to aggressive states under pressure	Specifies critical condition: proposes the “ego depletion threshold” as the pivotal trigger for critical state reversals, significantly enhancing predictive power and potential for intervention.

### 6.1 Addressing ambiguity in GAM

Whilst the General Aggression Model (GAM) emphasises individual situational interactions, it lacks a specification for a clear mediating mechanism. The SDA model fills this gap by identifying “ego depletion” as the core mediating variable, explaining how sustained pressure inevitably weakens inhibitory function through resource exhaustion.

### 6.2 Filling the physiological gap in the I^3^ model

The I^3^ Model proposes a tripartite interactionist framework but neglects underlying biological mechanisms. The SDA model integrates specific possible physiological pathways: including HPA axis activation, cortisol elevation, and inhibition of prefrontal glucose metabolism, thereby quantifying the biological substrate of dynamic pressure accumulation.

### 6.3 Innovating a “motivation-resource” dual-pathway explanation

This model represents the first integrative framework coupling meta-motivational dynamics (Reversal Theory) with resource limitations (Self-Control Theory). It explains the “why” of aggression through meta-motivational goal reconstruction, predicts the “when” based on the resource depletion critical point, specifies the “what” type determined by state combination, and informs the “how” of prevention. Based on the dynamic pathways delineated by the SDA model, we propose the following multi-level, actionable interventions:

Athlete level:

Pressure management: Train athletes to recognise high-pressure signals and establish personalised pressure threshold alerts.
Resource recovery: Implement brief mindfulness breathing exercises or rapid glucose supplementation strategies during identified high-depletion phases to mitigate ego depletion.
Motivational regulation: Utilise scenario simulation training to practise maintaining a conformist state or proactively switching to an adaptive mastery state under pressure.

Coach/team level:

Rotation strategy: Strategically schedule rest periods for core players during critical matches to prevent inhibitory failure from sustained resource depletion.
Psychological support: Establish process-orientated pre-match goals to mitigate telic pressure from outcome-focused demands.
Real-time intervention: Implement wearable devices tracking physiological markers to assess depletion likelihood.

Competition organisation level:

Referee sensitivity: Enhance conflict anticipation in high-pressure scenarios with preemptive interventions before depletion thresholds.
Rule adaptation: Consider graduated sanction scales during high-pressure phases whilst evaluating competitive equity implications.

Through an in-depth analysis of Suárez’s biting incidents, this study constructs the SDA model. It must be emphasised that the case selection itself dictates that the model’s primary contribution lies in theoretical heuristic value and precision, rather than statistical generalizability. Suárez’s behavioural pattern acts like a set of highly amplified experimental data, allowing us to precisely trace the complete psychological chain from pressure accumulation to behavioural loss of control. However, we acknowledge that a model built on a single case requires its external validity to be systematically tested in future research.

## 7 Study limitations and future directions

This study aimed to propose a novel stress-depletion-aggression (SDA) model through theoretical integration to elucidate unsanctioned aggression in elite athletes under extreme pressure. As a theory-building study, its primary contribution lies in providing a systematic analytical framework and a set of testable hypotheses, rather than offering final empirical conclusions. We clearly recognise that the validity and reliability of the proposed SDA model require rigorous testing in future empirical research.

Specifically, this study has the following limitations, which also constitute key avenues for future research:

### 7.1 Gap between theoretical derivation and empirical validation

The model was constructed primarily based on a retrospective analysis of the paradigmatic case of Luis Suárez. Although this case holds high theoretical value due to the extremity, repetition, and situational specificity of the behaviour, the model’s generalizability needs to be tested across broader athlete populations (e.g., different sports, cultural backgrounds, skill levels) and different types of aggressive behaviours.

### 7.2 Measurement of internal psychological states

Our analysis of Suárez’s level of ego depletion and meta-motivational states at the time of the incidents relied on theoretical inference from publicly available contextual information. Future research needs to adopt more direct methods to measure these internal states, such as using the Experience Sampling Method, wearable physiological devices (monitoring heart rate variability, galvanic skin response), and brief cognitive tasks (e.g., Stroop task) in real-game environments to assess the level of ego depletion in real-time.

### 7.3 Empirical testing and operationalization of the SDA model

To enable the SDA model to be tested and falsified by empirical research, we propose the following operational definitions for its core constructs and put forward a series of testable hypotheses:

#### 7.3.1 Operationalization and measurement of core variables

Stress can be operationalized as objective contextual variables, such as: match time remaining, score differential, match importance, discrepancy between personal performance and expected goals, accumulated number of fouls received, and controversial referee decisions. These variables can be directly quantified from match records and databases.

Ego depletion can be assessed using several proxy indicators. Physiologically, researchers can measure elevated salivary cortisol levels and reductions in heart rate variability (HRV). Behaviourally, performance declines on executive function tasks such as the Stroop task, Continuous Performance Task (CPT), or anti-saccade task administered during match intervals or in simulated scenarios provide evidence of impaired inhibitory control. Subjectively, brief self-report scales can capture participants’ perceived feelings of depletion. Combining these physiological, behavioural, and subjective measures offers a comprehensive evaluation of an individual’s ego-depletion state.

Meta-motivational state reversal can be evaluated through a combination of self-report and observational methods. Researchers can administer validated reversal theory state scales before and after matches or in simulated high-pressure scenarios to capture participants’ subjective state changes. In parallel, trained observers can code match footage for behavioural markers that signal specific states, such as frequent arguments with referees indicating a negativistic state or an excessive pursuit of physical dominance reflecting a mastery state. Integrating these psychological scales with systematic behavioural coding provides a robust assessment of meta-motivational state reversals.

Aggression requires establishing a clear behavioural taxonomy and coding system, strictly distinguishing between hostile and instrumental aggression, as well as sanctioned and unsanctioned behaviours, with clear definitions and recordings for rare but severe acts like biting.

#### 7.3.2 Testable hypotheses based on the SDA Model

Based on the above operational definitions, we propose the following core hypotheses to provide clear targets for future testing:

*H1 (Stress-Depletion Pathway)*: In high-pressure competitive situations, athletes’ physiological stress markers will be positively correlated with behavioural/neural indicators of ego depletion, and the mediating effect of this pathway will be significant.

*H2 (Depletion-Reversal Pathway)*: The degree of ego depletion will negatively predict the behavioural threshold for switching from a conformist state to a negativistic or mastery state.

*H3 (Motivation-Typology Pathway)*: Different types of stressors will be associated with specific meta-motivational state combinations and will predict the type of aggressive behaviour.

*H4 (Intervention-Prevention Pathway)*: Targeted resource recovery interventions implemented at key pressure points during a match will effectively delay ego depletion, thereby significantly reducing the probability of athletes engaging in unsanctioned aggression in the subsequent half.

### 7.4 Model applicability and boundaries

Although derived from an in-depth analysis of an extreme case involving an elite footballer, the core mechanism of the SDA model—stress input leading to ego depletion, which triggers meta-motivational state reversal and subsequent behavioural output—holds potential for cross-situational generalizability. It could be applicable to explaining similar loss-of-control behaviours in other contact sports and might even be extended to other domains requiring high-intensity self-regulation.

However, the model’s explanatory power also has clear boundaries. For non-elite athletes, factors like skill automaticity, baseline resource levels, and stress-coping strategies may differ, potentially requiring adjustments to the model’s parameters. In non-contact sports, the manifestation of aggression might shift from physical acts to equipment sabotage or self-directed aggression, and the associated meta-motivational state combinations might also differ. Furthermore, for aggressive behaviours driven by severe personality disorders or mental illnesses, which are frequent and lack specific situational triggers, the explanatory power of the SDA model might be superseded by more pathology-oriented theories. Future research should aim to test and calibrate this model across these diverse populations and contexts.

The baseline SDA model constructed in this study has not yet systematically integrated macro-level social-cultural factors. As mentioned above, variables such as team culture, coaching styles, and cultural background are likely to play moderating roles in the core pathways. Therefore, an important direction for future research is to employ cross-team, cross-cultural research designs to quantitatively test these moderating effects. For example, comparing teams coached by leaders with different styles, or examining differences in the SDA model’s path coefficients across leagues in different cultural contexts. This will contribute to building a more comprehensive, ecologically valid “bio-psycho-social model of aggressive behaviour.

Furthermore, our analysis is inherently subject to the challenges of retrospective interpretation. The primary data sources—media reports, match footage, and disciplinary records—carry an inherent risk of media framing bias, where the narrative surrounding Suárez’s actions may have been simplified or sensationalised. Our theoretical reconstruction of his internal psychological states (ego depletion, meta-motivational reversals) is necessarily inferential, as it is based on observable context and behaviour rather than direct psychological data from Suárez himself. Whilst we have striven to ground our inferences in established psychological theory and consistent behavioural patterns across multiple incidents, we acknowledge that alternative interpretations of his motivations remain possible in the absence of first-person data.

In summary, the primary theoretical contribution of the SDA model lies in establishing the first multi-level explanatory framework for aggression that integrates physiological, neural, and motivational perspectives. Its practical value resides in providing a scientific foundation for athletic cognitive training programs and evidence-based competition rule optimisation. Future empirical research is needed to test and refine the proposed SDA model and its hypotheses.

## Data Availability

The original contributions presented in the study are included in the article/supplementary material, further inquiries can be directed to the corresponding author.
